# ERAS with or without supplemental artificial nutrition in open pancreatoduodenectomy for cancer. A multicenter, randomized, open labeled trial (RASTA study protocol)

**DOI:** 10.3389/fnut.2023.1113723

**Published:** 2023-03-27

**Authors:** Luca Gianotti, Salvatore Paiella, Isabella Frigerio, Nicolò Pecorelli, Giovanni Capretti, Marta Sandini, Davide Paolo Bernasconi

**Affiliations:** ^1^School of Medicine and Surgery, University of Milano-Bicocca, Monza, Italy; ^2^HPB Unit IRCCS San Gerardo Hospital, Monza, Italy; ^3^General and Pancreatic Surgery Unit, Pancreas Institute, University of Verona Hospital, Verona, Italy; ^4^Unit of HPB Surgery, Pederzoli Hospital, Peschiera del Garda, Italy; ^5^Division of Pancreatic Surgery, Pancreas Translational and Clinical Research Center, San Raffaele Scientific Institute, Milan, Italy; ^6^Department of Biomedical Sciences, Humanitas University, Pieve Emanuele, Italy; ^7^Pancreatic Surgery, IRCCS Humanitas Research Hospital, Rozzano, Italy; ^8^Surgical Oncology Unit, Department of Medical, Surgical, and Neurologic Sciences, Policlinico Le Scotte, University of Siena, Siena, Italy; ^9^Bicocca Bioinformatics Biostatistics and Bioimaging Centre - B4, School of Medicine and Surgery, University of Milano-Bicocca, Monza, Italy

**Keywords:** ERAS, artificial nutrition, outcome, pancreatoduodenectomy, complication, randomized controlled trial

## Abstract

**Purpose:**

The role of supplemental artificial nutrition in patients perioperatively treated according to enhanced recovery programs (ERAS) on surgery-related morbidity is not known. Therefore, there is a need of a clinical trials specifically designed to explore whether given a full nutritional requirement by parenteral feeding after surgery coupled with oral food “at will” compared to oral food “at will” alone, within an established ERAS program, could achieve a reduction of the morbidity burden.

**Materials and analysis:**

RASTA will be a multicenter, randomized, parallel-arm, open labeled, superiority trial. The trial will be conducted in five Italian Institutions with proven experience in pancreatic surgery and already applying an established ERAS program. Adult patients (age ≥ 18 and < 90 years of age) candidate to elective open pancreatoduodenectomy (PD) for any periampullary or pancreatic cancer will be randomized to receive a full ERAS protocol that establishes oral food “at will” plus parenteral nutrition (PN) from postoperative day 1 to day 5 (treatment arm), or to ERAS protocol without PN (control arm). The primary endpoint of the trial is the complication burden within 90 days after the day of surgery. The complication burden will be assessed by the Comprehensive Complication Index, that incorporates all complications and their severity as defined by the Clavien-Dindo classification, and summarizes postoperative morbidity with a numerical scale ranging from 0 to 100. The H0 hypothesis tested is that he administration of a parenteral nutrition added to the ERAS protocol will not affect the CCI as compared to standard of care (ERAS). The H1 hypothesis is that the administration of a parenteral nutrition added to the ERAS protocol will positively affect the CCI as compared to standard of care (ERAS). The trial has been registered at ClinicalTrials.gov (number: NCT04438447; date: 18/05/2020).

**Conclusion:**

This upcoming trial will permit to establish if early postoperative artificial nutritional support after PD may improve postoperative outcomes compared to oral nutrition alone within an established ERAS program.

## Introduction

The enhanced recovery after surgery (ERAS) program is currently considered the gold-standard pathway for perioperative care in many types of operations (1) including pancreatoduodenectomy (PD) (2). The protocol is a bundle of interventions derived from the best evidence-based perioperative treatments aimed to accelerate patient functional recovery through the reduction of dysmetabolism and dyshomeostasis caused by surgery- and anesthesiology-related injury. In general, the implementation of ERAS generates a reduction of surgery-related complication, duration of hospitalization, and health care-related costs (1).

The intake of adequate qualitative and quantitative nutritional substrates is needed for appropriate tissue healing and recovery/maintenance of organ function after major surgery. To recover gut function and tolerate early postoperative oral feeding, many ERAS elements need to be implemented as they act in synergy (3).

PD is one of the most complex and challenging abdominal operations with a high rate of morbidity (4) and significant catabolic consequences. Moreover, the proportion of patients undergoing PD for cancer are at high nutritional risk or suffer some nutritional derangements at baseline in up to 80% of the cases (5). In addition, delayed gastric emptying (DGE) after PD is frequent (up to 50%) (6) compromising the regular resumption of oral food with the risk of developing postoperative malnutrition.

According to expert opinions (7), artificial nutritional support should be implemented early postoperatively in malnourished patients, in those patients at high risk of developing malnutrition, in those who develop complications affecting oral feeding tolerance, and in well-nourished patients who do not tolerate at least 50% of their caloric and protein requirement by postoperative day 7 for any reason. Accordingly, most of the patients bearing pancreatic cancer and undergoing PD should receive some form of artificial nutritional support after the operation. Conversely, ERAS pathways promote oral food “at will” early after surgery and consider an artificial nutritional support only in selected cases (8). Furthermore, there are no convincing data on whether attaining adequate nutritional needs can be accomplished only by progressive increase of oral food intake. A study reported (9) that, the mean daily calorie and protein intake in the first 2 weeks were similar between the ERAS group and the patients managed conventionally. Anyhow, the results revealed that the total energy goal through oral feeding was not reached in both groups. Other studies did not analyze or reported incomplete data on tolerance to early postoperative oral feeding (EOF) after PD (10, 11). Robertson et al. (12) described compliance rates of 82% for resumption of oral fluids and 86% for tolerance of solid diet. Conversely, in another large study (13), postoperative oral liquids were tolerated by 55% of the patients and solid food in 53%, but compliance dropped substantially in patients with a complicated postoperative course. Thus, the available evidence suggests that using only oral feeding (food “at will”) within an ERAS protocol may be only partially adequate to achieve the nutritional needs after PD.

Given the lack of strong evidence, there is a need for a randomized clinical trial specifically designed to explore the extent to which reaching full nutritional requirements by adding parenteral feeding in the first days after surgery within an established ERAS program impacts on postoperative morbidity compared to oral food “at will” alone.

## Study design and management

RASTA will be a multicenter, randomized, parallel-arm, open labeled, superiority trial.

The trial will be conducted in five Italian Institutions with proven experience in pancreatic surgery and an established ERAS program.

THE RASTA trial will be managed and coordinated by the School of Medicine and Surgery of the Milano-Bicocca University and the HPB Unit of the IRCCS San Gerardo Hospital, Monza, Italy. The coordinating center will also be responsible for treatment allocation and monitoring, and statistical analysis with the support of the Centre of Biostatistics for Clinical Epidemiology of the Milano-Bicocca University.

The trial has been registered at ClinicalTrials.gov (ID: NCT04438447; date: June 16, 2020) and approved by the Italian Drug Agency (AIFA; number: EudraCT 2020–005483-66; date: September 13, 2021).

### Pre-trial training

Before starting patient enrolment, multiple meetings will be organized to accomplish:

- Correct definition of eligibility, inclusion and exclusion criteria.
- Agreement on definition of postoperative complications.
- Training on randomization process and patient instruction on treatment arms.
- Accordance on ERAS elements (described in Table 1).
- Training of outcome assessors to record the occurrence of the primary and secondary endpoints. Each participating center will nominate two independent outcome assessors. The assessors will be trained by the single center principal investigator on the definition of complications during a dedicated pre-trial face-to-face meeting according to a modified Delphi method. In case of discordance on the assignment of the endpoint, a third expert will intervene to solve the dispute and classify the patients as complicated or not. Outcome assessors will be blinded to treatments.
- Training on how to fill out correctly the case report form.

**Table 1:** ERAS items implemented in both groups.

Item	Yes	No
Preoperative counseling	X	
Prehabilitation		X
Preoperative biliary drainage		X
Preoperative stop of smoking and alcohol consumption	X	
Preoperative nutrition		X
Perioperative oral immunonutrition		X
Preoperative fasting		X
Preoperative carbohydrate loading	X	
Pre-anesthetic medication		X
Anti-thrombotic prophylaxis	X	
Antimicrobial prophylaxis and skin preparation	X	
Epidural analgesia	X	
Opioid-sparing analgesia	X	
Wound catheter and transversus abdominis plane block	X	
Minimal invasive surgery		X
Nausea and vomiting prophylaxis	X	
Avoiding hypothermia	X	
Nasogastric intubation		X
Goal-directed fluid therapy	X	
Perianastomotic drainage	X	
Somatostatin analogues		X
Postoperative multimodal analgesia	X	
Postoperative glycemic control	X	
Urinary drainage early removal	X	
Stimulation of bowel movement		X
Early and scheduled mobilization	X	
Audit	X	

### Patient eligibility

Adult patients (age ≥ 18 and < 90 years of age) scheduled for elective open pancreatoduodenectomy for any periampullary or pancreatic cancer.

### Inclusion criteria

- Patients must be willing to participate in the study and able to provide written informed consent form prior to any study activity.
- Preoperative normal renal function, blood electrolytes (sodium, potassium, chlorite) and coagulation tests (PT, PTT).

### Exclusion criteria

- American Society of Anesthesiologists (ASA) physical status classification >3
- Preoperative severe malnutrition (Weight loss >15% with respect to usual weight in the last 6 months, according to the new GLIM criteria) (14).
- Ascites
- Any proven hypersensitivity reaction to parenteral nutrition (PN) components
- Palliative surgery
- Early postoperative administration of enteral nutrition *via* a naso-enteric or jejunostomy feeding tube placed during surgery.

### Screening and randomization processes

After being screened for inclusion and exclusion criteria, patients or their legal representative will be asked to sign a written informed consent. After enrolment in the study, patients will be randomly allocated into two arms. All reasons for exclusion after screening will be recorded.

Patients will be randomly allocated to ERAS or ERAS plus PN at 8:00 PM of the day of surgery, or at 8 AM in the morning of postoperative day one if the operation was concluded after 8 PM. Randomization will be performed by a computed-generated permuted-block sequence. A specific code will be generated for each center to achieve equivalent grouping. The allocation ratio will be 1:1 with a block size of 4. Randomization will be stratified by neoadjuvant chemo- or chemoradiation therapy and center. Randomization will be competitive among centers.

Surgeons and patients will not be blinded to treatment arm. Masking to allocation will be impossible to achieve for the study nature and design.

Patient chart evaluation and data entry for outcome recording will be done by trained assessors (selected in each center) and not directly involved in patient care and thus masked to patient allocation. Clear information on patient allocation will not be released to any hospital personnel with exception of ethical committee members under specific request.

### Study duration and definition of termination

The expected duration of enrolment is approximately 2 years.

The study will be considered as terminated when the last enrolled patient will have completed the 90-day follow-up after the date of surgery.

### Study intervention

Patients randomized in the treatment arm will be treated with a full ERAS protocol that establishes oral food “at will” plus parenteral nutrition (PN) from postoperative day 1. A ready-to-use, all-in-one, 3-bag compartment peripheral parenteral solution (mOsm <800) (Olimel N4E®, Baxter Italia, SpA) containing carbohydrate, lipids and proteins will be infused to deliver 20/25 total Kcal/kg/day for a total of 5 days after the operation with the addition of I.V. supplementation of vitamins (one vial/day) (Cernevit®, Baxter Italia SpA).

In case of occurrence of any complication impairing the full or partial recovery of oral food, the treatment will be continued or switched to tube enteral feeding until clinically indicated.

Administration of parenteral nutrition will be through a peripheral vein with a rate of delivery that is calculated based on patient body weight. The total volume of parenteral nutrition will be the result of the calculation of the amount of prescribed calories, multiplied for the patient body weight.

### Control arm

Patients randomized in the control arm will be treated with a full ERAS protocol that establishes oral food “at will” after the operation. In case of occurrence of any complication impairing the full recovery of oral food within postoperative day 5, patients may receive parenteral or enteral nutrition as clinically indicated.

### Procedures common to both arms

Patients of both groups will be treated according to the ERAS Society guidelines for perioperative care for PD (Table 1) (8). Blood glucose ≥180 mg/dl will be treated with insulin injection (either subcutaneous or by continuous IV infusion). Open PD technique will be chosen by the participating centers according to their standards.

### Study plan

Study plan and schedule of assessment are summarized in Table 2.

**Table 2:** Study plan and schedule of assessment.

Visit	V pre	V1	V2		V3	V4	In hosp FU	Dis	FU
Time interval	Pre random	Basal at random	Intraoperative	Day 1	Day 3	Day 5	Once a day during index hospitalization	Discharge	3-mo. follow-up (from the day of surgery)
Informed consent	X								
Demographics	X								
Height	X								
Weight	X							X	X
Body mass index	X								
Physical examination (every day until discharge)	X			X	X	X	X	X	X
Patient history	X								
Inclusion criteria		X							
Exclusion criteria		X							
Blood pressure		X	X		X	X	X		
Heart rate		X	X		X	X	X		
ECG		X							
ICU admission							X		
Lab tests*		X		X	X	X	X	X	
Need of insulin			X	X	X	X	X	X	X
NRS-2002		X							
Death			X	X	X	X	X	X	X
Safety endpoints				X	X	X	X	X	X
Blood loss			X						
Duration of surgery			X						
Surgical details			X						
Fluid balance			X	X	X	X	X		
Histology			X					X	
Analgesia			X	X	X	X		X	
Discharge criteria						X	X		
Complications (CCI)				X	X	X	X	X	X
Drug administration (DAY 1-5)				X	X	X			

*Urea, Creatinine, Complete blood count, PT, PTT, Glucose, Na, K, Cl, prealbumin, albumin, glycate haemoglobin, bilirubin, CRP.

### Ethical aspects

The study has been approved by the Competent Authority (AIFA) the Ethical Committee of all participating centers. The local Ethical Committee, as coordinating center, provided the “not emendable judgement” according to the Italian legislation (approval number: 3467; date: February 11, 2022).

## Outcomes

The primary endpoint of the trial is the complication burden within 90 days after surgery. The complication burden will be assessed by the Comprehensive Complication Index (CCI) (15), that incorporates all complications and their severity as defined by the Clavien-Dindo classification, and summarizes postoperative morbidity with a numerical scale ranging from 0 (no complication) to 100 (death).

### Hypothesis tested

#### H0 hypothesis

The administration of a parenteral nutrition added to the ERAS protocol will not affect the CCI as compared to standard of care (ERAS).

#### H1 hypothesis

The administration of a parenteral nutrition added to the ERAS protocol will positively affect the CCI as compared to standard of care (ERAS).

Secondary outcome measures will be:

- Actual daily calories delivered by PN.
- Rate of unplanned artificial nutrition (for control group).
- Rate and severity of complications at 90 days after discharge.
- Rate of surgical site infections (16)
- The rate and severity of postoperative pancreatic fistula (17)
- Rate and severity of DGE (18)
- Rate and severity of hemorrhage (19)
- Length of stay (LOS) based on predefined criteria
- Actual LOS
- Rate of reoperation.
- Rate and duration of intensive care treatment.
- Rate of hyperglycemia (blood glucose >180 mg/dl)
- Use of insulin (subcutaneous bolus or continuous infusion)
- Δ plasma prealbumin levels (baseline, postoperative day 1 and 6)
- Use of morphine
- Readmission rate
- Body weight (90 days)
- 90-day mortality

Any attending surgeon will decide the day of discharge according to his individual clinical judgement. However, LOS will be also calculated by the achievement of pre-specified discharge criteria (full patient mobilization, pain controlled by oral therapy, full tolerance to oral feeding). In particular, a visual analog pain scale ≤2 must be achieved for safety discharge.

Post-discharge follow-up will be accomplished by weekly outpatient visits. Also telephone interviews will be allowed to monitor patient health state, but in case of warning signs or symptoms of a complication, patients will be asked to refer to the hospital where the operation was performed for further clinical evaluation.

### Safety issue

Adverse events:

- the number of patients not reaching tolerance to oral feeding within 7 days after surgery
- the number of patients needing insulin therapy.
- the number of patients requiring electrolyte corrections.

## Statistical planning

The sample size of 120 patients per group is necessary to provide an 80% power to detect at least a 30% reduction in the CCI, which is expected to be around 23 (median) (IQR 21–31) or mean 27 (±20 SD) in complicated patients of the control group. The hypothesized reduction of 30% is based on sound clinical relevance meaning that such reduction will have a consistent and significant impact of the postoperative course with advantages on well-being, quality of life, shorter length of hospitalization and a relevant reduction of heath care burden and resources. The median CCI of 23 is retrieved from a previous publication (20). The rate of complication in this type of surgery is expected to be approximately of 60%.

A Mann–Whitney test is considered, type I error rate is fixed at 5% (two tails) and an expected drop-out of 10% is taken into account.

For the binary end-points, the relative risk (RR) with the corresponding 95% confidence interval, comparing the two groups, will be estimated. For the primary end-point, also the risk difference (RD) will be computed. For the numerical end-points the difference in the location parameter (i.e., median pairwise difference) between the two groups with the corresponding 95% confidence interval will be computed. Fisher test and Mann–Whitney test will be adopted to evaluate univariate associations. Incidence of complications over time in the two groups will be described according to the Nelson-Aalen cumulative hazard estimator also accounting for multiple events per patient. The incidence in the two groups will also be compared by computing the incidence rate ratio (with 95% confidence interval). This analysis will be performed both considering all complications and only major complications.

A multivariate quantile regression model (focused on median, 25^th^ and 75^th^ percentiles) will be used to identify factors associated with the primary endpoint and to evaluate the effect of treatment adjusting for possible residual confounding. Logistic regression will also be used to model the probability of CCI >23. Using these regression models, the effect of PN over controls on the CCI will be also investigated within pre-specified subgroups to account for possible effect modification. The pre-specified risk factors for this analysis will be:

- Nutritional risk screeing-2002 (≥3)
- Body mass index (> 30)
- Sex (male)
- Age (>70 years)
- Charlson comorbidity index (>4)
- ASA score (=3)
- Blood loss (≥500 mL)
- Duration of surgery (>360 min)
- Biliary stenting
- Diabetes
- Pylorus-preserving PD (vs. Whipple)
- Pancreatic ductal carcinoma (vs. others)
- Fistula risk score (≥7)
- ERAS overall compliance (>70%)

All analyses will be done based on the principles of “intention-to-treat” and “per-protocol” and performed with the R software.

### Study stopping rules

An ad interim analysis will be done at the achievement of 50% of the study power (120 patients in total). The study will be stopped only in case of an increase over 30% of the median CCI in either groups. Study will be stopped immediately in case death or, a life-threatening experience (that is, immediate risk of dying) related to the use of PN, or a persistent or significant disability/incapacity will exceed 5% of the enrolled population. A Data and Safety Monitoring Board will oversee and monitor the trial to ensure participant safety and the validity and integrity of the data.

## Data collection and management

All data will be collected into an electronic database with a double entry to assure consistency of records. In case of missing or implausible data, queries will be mailed to the participating centers to obtain integrations or corrections. Data collectors will be blinded to allocation.

The patient first and last name and date of birth will be omitted according to the Italian legislation on privacy. Subject identification will be carried out only by the randomization code.

All data will be collected into a dedicated excel spreadsheet. This electronic registry will be identical for all centers and each center will have their own dataset. The excel spreadsheet will be protected by a password possessed by the assessor.

### Case report form

The following baseline patient-related parameters will be recorded:

- Age (years)
- Sex
- Weight (kg)
- Height (m)
- Body mass index (kg/m^2^)
- Nutritional risk score-2002
- Percent of weight loss in the 6 months prior to surgery
- Charlson comorbidity index
- Diabetes
- Jaundice
- Biliary stenting
- Routine laboratory test (albumin, preabumin, bilirubin, hemoglobin, creatinine, HbA1c, CRP)
- Primary disease with indication to surgical resection
- ASA score
- Neoadjuvant treatments

The following intraoperative parameters and events will be recorded:

- The day of operation
- Type of surgical procedure (PPPD, Whipple)
- Type of pancreatic anastomosis (gastric, jejunal)
- The level of intraoperative contamination (clean; clean-contaminated; contaminated; dirty)
- Use of epidural analgesia, TAP block, subfascial catheter
- Intraoperative hypothermia (defined as body temperature < 35.5°C for more than 30 min)
- Estimated blood loss (mL)
- Volume of IV fluid infusion
- Intraoperative blood transfusion
- Fluid balance (in and out difference)
- Duration of operation (minute)
- Main pancreatic duct diameter
- Pancreas texture (soft, intermediate, hard)
- Fistula risk score

After the operation the following parameters and events will be recorded:

- Capillary blood glucose levels (every 6 h for 5 consecutive days)
- Any administration of insulin (for blood glucose ≥180 mg/dL)
- Occurrence of a complication (90-days)
- Type of complication
- The complication burden according to CCI (90-day)
- The severity of complication according to Clavien-Dindo classification
- The need of reoperation, reason and postoperative day
- The need of unplanned intensive care treatment and the duration (days)
- The day of canalization to gas and stools
- The day of resumption of oral feeding
- The need and duration of artificial nutrition (for the control arm)
- Potential hospital discharge according to predefined criteria
- The actual day of hospital discharge
- Disease staging
- Readmission rate (30-day)
- Mortality rate (90-day)

## Discussion

The use, timing of initiation, and route of delivery of artificial nutrition after PD is still a matter of debate for the conflicting evidence and the difference in study design. One randomized trial, showed that in patients submitted to PD and kept “nil by mouth” for 10 days after the operation, immediate parenteral feeding was associated with less complications when compared to progressive tube enteral nutrition (21) suggesting that the achievement of an immediate and full nutritional goal may be protective on the risk of morbidity. One systematic review (22) compared the outcomes of 5 feeding routes after PD and reported no difference in terms of safety and efficacy. A recent meta-analysis by Tanaka et al. (23) advocated that routine enteral nutrition after PD was associated with a lower incidence of infectious complications and a shorter length of hospital stay than non-enteral nutrition. Percutaneous tube feeding had a lower incidence of infectious complications and a shorter hospital stay than parenteral nutrition whereas naso-jejunal tube feeding was not associated with better postoperative outcomes. Thus, the authors concluded that as a supplement to regular oral diet, routine enteral nutrition, especially *via* a percutaneous enteral tube, may improve postoperative outcomes after PD.

The results of another randomized controlled trial (RCT) (24) on patients who underwent pylorus-preserving PD suggested additional early tube enteral nutrition did not affect the frequency of DGE and did not offer any further clinical advantages over early oral feeding. However, in persisting DGE, better outcomes were achieved when artificial nutrition, either parenteral or enteral, was started within 10 days of operation (25).

After the development of a clinically relevant pancreatic fistula, the use of enteral tube feeding was not superior to oral nutrition in terms of 30-day fistula closure rate. Compared with enteral feeding, oral feeding significantly reduced hospital costs and duration of stay (26).

Despite not specifically designed for patients undergoing PD, two recent large RCTs provided conflicting results on the need of early artificial nutritional support after major abdominal surgery. Zhang et al. (27) randomized patients at high nutritional risk, to immediate vs. gradual advancement to goal of enteral tube nutrition. The first group received 100% of the caloric requirement on postoperative day 3, while the other received 40% progressing to 80% of target on day 7. The results showed that immediate enteral feeding was non-inferior to gradual advancement in regards to infectious complications. However, immediate feeding was associated with more gastrointestinal intolerance events. The other trial (28) randomized 230 patients at high nutritional risk and poor tolerance to tube enteral nutrition, to receive supplemental PN early (on day 3) or late (on day 8) after surgery. The early group had significantly fewer nosocomial infections compared with the late group (8.7% vs. 18.4%; *p* = 0.04). No significant differences were found between the early and late group in the number of noninfectious complications. The authors concluded that early supplemental PN appeared a favorable strategy for patients with high nutritional risk and poor tolerance to EN.

In 2022, Joliat et al. (29) published the protocol of a multicenter, open-label, RCT for patients undergoing PD with a nutritional risk screening ≥3 in a setting of full ERAS strategy. Patients will be randomized to receive either early enteral nutrition (intervention group) or oral nutrition (control group) after the operation. Patients in the intervention group will receive tube enteral nutrition since the first night of the operation and the infusion will be increased daily if tolerated. The primary outcome will be the CCI at 90 days after surgery.

Differently from the above study design, we opted for parenteral nutrition instead of enteral tube feeding. The rational of giving parenteral feeding has been based on the ability of this therapy to provide the exact amount of calories and protein since the very beginning of administration. As opposite, tube enteral nutrition needs at least 4/5 days to reach the caloric target or even more depending on tolerance (30).

These two upcoming trials will allow to establish if early postoperative artificial nutritional support after PD may improve postoperative outcomes compared to oral nutrition alone within an established ERAS program. Moreover, the results might be useful for a potential updated version of the International Study Group on Pancreatic Surgery recommendations (7) on nutritional therapy in pancreatic surgery.

## Ethics statement

The studies involving human participants were reviewed and approved by Monza e Brianza ethical committee. The patients/participants provided their written informed consent to participate in this study. Written informed consent will be obtained from the individual(s) for the publication of any potentially identifiable images or data included in this article.

## Author contributions

LG, SP, MS, IF, NP, GC, and DPB contributed to conception, design of the study, and wrote sections of the manuscript. LG, SP, and MS organized the database. DPB will perform the statistical analysis. LG wrote the first draft of the manuscript. All authors contributed to manuscript revision, read, and approved the submitted version.

## Funding

The Italian Society of Clinical Nutrition and Metabolism (SINPE) has provided funds for insurance cost. SINPE will need to approve the final version of the manuscript. Baxter Italia SpA, will provide, as donation, the parenteral bags and the vitamin vials for the 5-day duration of treatment of the experimental group. Baxter Italia SpA will not have any role in the study design and data analysis and interpretation.

## Conflict of interest

The authors declare that the research was conducted in the absence of any commercial or financial relationships that could be construed as a potential conflict of interest.

## Publisher’s note

All claims expressed in this article are solely those of the authors and do not necessarily represent those of their affiliated organizations, or those of the publisher, the editors and the reviewers. Any product that may be evaluated in this article, or claim that may be made by its manufacturer, is not guaranteed or endorsed by the publisher.
